# Supplementary material for: Evaluation of post-operative complications, outcome, and long-term owner satisfaction of elbow arthrodesis (EA) in 22 dogs
Source: PLoS One. 2021 Jul 30;16(7):e0255388. doi: 10.1371/journal.pone.0255388 (PMC8323925; doi:10.1371/journal.pone.0255388)
Supplement: Attachment — Submitted filename: Response to Reviewers.docx [file pone.0255388.s001.docx]

Response to reviewers:

Thank you both for taking the time to review, and comment about our manuscript.

We hope that with this version all comments were addressed.  Please let us know if any additional changes are needed.

Sincerely

Dr. Dinwiddie and authors

Reviewer #1:

While the content of the manuscript was generally straight-forward, the scientific writing style needs much improvement, particularly pertaining to grammar. The authors should consider having the paper professionally edited prior to the next submission.

-The manuscript has been re-written and professionally edited by experienced authors multiple times prior to re-submission making direct correction to grammar, sentence structure, and writing style.

Additionally, it is not appropriate to report 'rates', 'common/uncommon' occurrences, and '%' when case numbers are so low (e.g. line 226 the most common major complication was pin migration (2/4)). Simply report the numbers as they are.

-The sentence structures of the manuscript have been addressed to reflect numbers simply as reported. There are only 2 previously reported articles regarding EA which have are referenced in the manuscript. This lack of information regarding EA is noted starting in line 66

There are over-reaching and misleading conclusions, which could be attributed to the lack of relevant data. Most importantly: reporting that most dogs had 'good to excellent quality of life' and 'high satisfaction' as one of the most relevant findings is highly misleading for this population. Indeed, most of the dogs had moderate or severe lameness on long term follow-up. The most striking case in point: (line 264-265)- one dog required a mobility cart due to lack of any weight-bearing on the arthrodesis limbs, yet the owners report good satisfaction and quality of life from the arthrodesis!! The authors should consider classifying outcomes as "full", "acceptable", "unacceptable" (per Cook et al., Vet Surg 2010) based on the questionnaire results. For instance, this reviewer would suggest outcomes of dogs with 'moderate' or 'severe' overall limb disability would be 'unacceptable'. Note that, per Cook et al Vet Surg 2010, it is possible to have a satisfied owner yet an unacceptable outcome. On a similar note- how is it possible to diagnose a 'mechanical' lameness by telephone conversation?

-The conclusions have been edited to reflect that while arthrodesis can be achieved via multiple fixation methods, care should be taken in performing this procedure because all fixation groups had complications. Over-reaching statements have been removed and/or edited to reflect that. In addition, we have added statements discussing Cook et al’s paper reflecting that there was case with satisfied owners yet unacceptable outcome (lines 395-399). Statements were also added to discuss mechanical lameness at 8 week surgical rechecks (lines 202-208) and that while mechanical lameness is expected, without physical exams, it is not possible to confirm that the lameness is indeed mechanical in nature (lines 355-367)

The case numbers within groups are too low to draw any major conclusions from comparisons between groups - e.g. only 3 dogs with ESF had follow-up. Therefore, 'recommendations' (e.g. cannot recommend ESF) should be tempered.

-Statements singling out any one type of fixation have been removed as all types of fixations had complications.

While the manuscript title suggests the case series focuses on complications, there is a clear intent to report overall outcomes as well. Therefore, 1) the title must be adjusted, and 2) more information regarding outcomes should be provided; namely- (when) was union of the arthrodesis documented for each case?

-Title has been edited Evaluation of post-operative complications, outcome, and long-term owner satisfaction of elbow arthrodesis (EA) in dogs: 22 cases (2009-2019)

In addition, we have added dialogue regarding post-operative evaluations as well as information regarding arthrodesis union (lines 161-162)

Line 43: Conclusions: major complication rate was high for all groups. Why single out ESF if final result not obviously different?

-Statements regarding conclusions have been edited to reflect that while complication rates were high for all fixation types, arthrodesis is achievable in all fixation types as well (lines 46-47)

Line 51-53: not sure how this is a ‘paradigm’; suggest deleting this sentence.

-sentence deleted

Paragraph starting line 65: Please report how many cases of elbow arthrodesis have been previously described. This will give better context for the relevance of this manuscript.

-Paragraph starting line 63 discusses infrequency of EA reporting to give relevance of this manuscript (One paper written in the last 10 years)

Line 86: why exclude these methods?

-the methods of fixation that the authors wished to include in this study were bone plate and ESF which are the most common. Any other form was eliminated for continuity purposes and so that the categories of EA could be maintained.

-Cases included were those with EA performed by plate fixation or ESF and reflected in the abstract lines 26-30

Line 120: What were the outcomes of the non-grafted cases? Grafting is a fundamentally important aspect of arthrodesis

-Paragraph starting line 163 discussed outcome of non-grafted cases

Line 137-138: Provide the scales for satisfaction (excellent, good, fair, poor)
-Scales have been added for all evaluations (surgical and owner) in lines 92-103

Line 154: please ensure none of the cases have been described in other papers; or otherwise disclose the information
-None of these cases have been previously reported. The other 2 papers regarding EA are referenced in the manuscript

Line 159-165: Redundant statements (already mentioned in the methods); delete
-Redundant information and statement have been removed and/or edited

Table 2 is not easy to read; consider revising to chart form.
 -Table 2 was revised into individual tables (Table 2, 3, and 4) to reflect responses to individual fixation types. The owners responses to the question on the left hand is written next to the possible options in parentheses to the right

Line 244 vs line 253: range is 1-9 years yet 2 dogs were euthanized < 1 year postoperatively?

-range was updated to reflect correct time frame (7 months-9 years)

Line 285-288: cut and pasted out of results; please rephrase
 -redundant information was removed and results section restated

Line 291-295: it is hard to agree with the logic here based on the data provided (i.e. that minor complications will lead to major complications).
-Paragraph has been re-written to discuss infection associated with these fixations as we cannot conclude that one minor complication leads to a major. Discussion thus surrounds the SSI portion of complications in paragraph starting 331

Paragraph starting line 296: this paragraph should discuss SSI occurrence for all groups, yet only ESF is discussed.

-Paragraph starting 331 edited to discusses SSI. In all forms of EA fixation, not just ESF

Line 311: How is it known that the lameness was mechanical, since outcomes were defined by telephone conversations with owners?. There is no mention of circumduction in the results?

-Information regarding surgical evaluation at 8 weeks post operatively has been added to include mechanical lameness as well as circumduction (lines 63-68). Mechanical lameness is expected with this procedure; however, without performing PE, we cannot confirm that on long term evaluations with the owners via phone call if the lamenesses were indeed mechanical. This is reflected in discussion starting lines 355-360.

Paragraph starting line 321: Is this supposed to be the limitations paragraph? There are many more limitations than the variable approaches taken i.e. incomplete/inaccurate records, lack of veterinarian assessed follow-up, highly heterogenous population etc etc.
-Limitations paragraph has been re-written to reflect a more conclusive list starting line 406

Paragraph starting line 333: The arguments here are based on extremely weak data. Indeed, from the table, it is not obvious that older animals had more complications or poorer outcomes.

-Paragraph was removed as argument was weak and not founded on information gathered for this manuscript

Line 348: If you are recommending a validated assessment, why was one not used??

- We used a modified version of the previously reported questionnaire as a means to keep data consistent. The other validating system (i.e. force plate analysis) would be useful post operatively, but due to the retrospective nature of this study, we could not conduct that type of analysis.

Line 258: "Good use of the operated limb" in the 'majority of patients' is simply not true; 10/14 dogs had moderate or severe limb disability; only 2/14 dogs did not have lameness at walk and run

-10/14 dogs had mild to moderate lameness long term which is an improvement for these patients from pre-operatively. Again, mechanical lameness is expected so we anticipate lameness with some of these cases. This should be more evident now that the Table 2 has been broken down into individual tables to reflect the long term outcome of patients.

Line 359-360: Where in the results is this shown? i.e. that ESF group had lacked improvement post-operatively and had lowest satisfaction (2 out of 3 owners were actually satisfied)?

-ESF group did have a good outcome which is now reflected in the ESF complications and outcome section begin line 257. Outcome for each fixation type has been re-written and added to the respective section

Please consider adding a paragraph in the discussion on the paradox between satisfied owners vs lack of good limb use. One possibility is that the owners were simply happy that their dog was not amputated.

-Paragraph starting 383 was added to discuss this paradox

Reviewer #2:

I struggled to follow certain parts of it as it was quite contradictory. For example, how can dogs have an excellent quality of life, yet be moderate to severely lame? Including one which had a mobility cart? The two seem somewhat contradictory to me. I think I understand what you mean, but I think it needs some quantification.

-Paragraph starting line 383 was added to reflect that there can be pleased owners that perceive a good quality of life for their pet despite that they have an unacceptable outcome.

I think I would also be tempted to edge on the side of caution with your conclusions, as although numbers are relatively large for this field, overall they are quite small.

-Conclusions have been tempered and re-written to reflect the data given here, that all fixation types can result in complete arthrodesis but all groups had complications; therefore, the conclusions are merely cautionary in nature about EA as a procedure regardless of fixation type

The title is also a little misleading (wrong word but couldn’t think up a better one). You have a lot on clinical final outcomes, so that maybe better in the title too?

- Title has been edited Evaluation of post-operative complications, outcome, and long-term owner satisfaction of elbow arthrodesis (EA) in dogs: 22 cases (2009-2019)

Line 54-57- its not the patients which have the difficult choices to make- it’s the owners!

-The Abstract and Introduction have both been revised and include discussion of treatment of cases with end stage elbow disease and what options are available for owners.

Line 85- how was quality of life assessed?

-Quality of life was one of the long term questionnaire questions and a subjective measurement by the owners meant for them to assess how their pet was doing at that point long term (Table 2, 3, 4).

Line 86- why were these methods excluded? Is there a specific reason?

-The line was re-written- the cases included were cases that had EA performed by either LCP, DCP, or ESF.

Line 106- . In total, twenty-two EA procedures were included in this study (Add in comma)

-The manuscript was professionally edited again and hopefully addressed most of the grammatical errors. This sentence was re-written with the comma added

Line 120- can you mention the outcomes of the cases? Particularly the grafts as these can sometimes be tricky to take

-Case outcomes have been included in the paragraph regarding each respective fixation type (i.e. DCP complications and outcome). Table 2 had been broken down to reflect each fixation type, so are now Table 2, Table 3, Table 4. In addition, images of each fixation type have been included as well.

Some of the results are included already above, please consider revising

-The materials/methods and results sections have been revised to better include information in correct sections.

I struggled to follow table 2- this may be easier as a figure, or with more explanation.

-Table 2 was broken down to reflect owner responses to individual fixation types and are now Table 2, Table 3, Table 4

Line 291- is a major complaint not just a progression of a minor one? I can see what you are saying here but its unclear. Perhaps a major complaint masks a minor?

-Both major and minor complications have been revised in both Table 1 as well as the text to better clarify what is minor vs major.

Line 303- To the authors knowledge, there is no published infection rate of (add in comma)

-This paragraph has been edited to discuss infection in all types of fixation not only ESF in paragraph beginning 331

Line 311- hpw do you know that it is mechanical lameness? Unlikely that the owner would pick this up.

- Statements were added to discuss mechanical lameness at 8 week surgical rechecks (lines 202-208) and that while mechanical lameness is expected, without physical exams, it is not possible to confirm that the lameness is indeed mechanical in nature (lines 355-367)

Line 321- the limitations are far greater than those explained, including inaccurate records, different vets, poor follow up etc

-The paragraph regarding limitations has been edited and includes more limitations than previously discussed. This is discussed in paragraph starting at line 406

Line 333- I have concerns about some of the arguments here as the data isn’t that strong- maybe have a read over it and consider rewording it?

-The discussion portion was re-written to take into consideration that due to the low numbers of cases, some overarching conclusions couldn’t be made. This paragraph was reworded with specific changes to the arguments.

Line 359- isn’t the ESF group the lowest improvement group post op?

-The ESF group had the most complications associated with it but only 1 patient had low improvement. This section was re-written to reflect complications and outcome for ESF lines 258-294
