# Supplementary material for: Evaluation of post-operative complications, outcome, and long-term owner satisfaction of elbow arthrodesis (EA) in 22 dogs
Source: PLoS One. 2021 Jul 30;16(7):e0255388. doi: 10.1371/journal.pone.0255388 (PMC8323925; doi:10.1371/journal.pone.0255388)
Supplement: Attachment — Submitted filename: Response to Reviewers (2).docx [file pone.0255388.s002.docx]

Response to reviewers:

Thank you both again for taking the time to review, and comments on our manuscript.

We hope that with this version all comments were addressed. This manuscript is required to obtain ACVS credentials by August 1, 2021.  Please let us know if any additional changes are needed prior to acceptance.

Sincerely,

Dr. Dinwiddie and authors

Reviewer #1: Thank you for the revisions, the manuscript is improved.

General comments:

Do not use the word ‘patient’ unless referring to human subject. Use ‘dog’ or ‘case’ instead.

-The term ‘patient’ was replaced with either dog or case throughout the manuscript

Delete all % values- the numbers are too small in this study.

-Percentage values were removed from the manuscript with the exception of the discussion of infection rates for this study. The statement of “small sample size” was added to the discussion paragraph to specifically recall that sample size plays a factor in these infection rates

The results and discussion are very lengthy and could be condensed further.

-Both the results and discussion have had portions removed or revised to condense the material.

Specific comments:

Line 36,37: “Complications were reported in X/X, Y/Y, and Z/Z for the DCP, LCP, and ESF groups, respectively”

-This line has been reworded as suggested above

Do not jumble methods with results in abstract – i.e. move sentence on line 37 to methods area

-Sentence removed from abstract and stated within methods section

Abstract conclusion: Yes it can be done, but persistent lameness is expected and complication rate is high.

-The concluding sentences were combined to read better

Line 54: amputation is a radical surgical intervention – delete ‘amputation’

-‘amputation’ was deleted

Line 63: Do not begin sentence with acronym

-Changed EA to Elbow Arthrodesis

Line 64: what is meant by ‘the most recent literature’?

-Defined and edited to say ‘in the past 10 years of literature’

Line 70: Something is missing between the last two paragraphs of the introduction. Need to provide some comment about advent of new implants i.e. locking plates that might influence success rates.
-Added paragraph addressing available implants

Line 78: acronym not used for EA

-Changed elbow arthrodesis to ‘EA’

Line 93: Outcome was based on evaluations/radiographs at what minimum time frame?

-Added ‘average of 9 weeks post operatively’. The full timeframe range is stated in the results section

Line 97: the time range provided belongs in the results.

-Moved timeframe to results section

Line 134: why 9/9? The number of cases is already listed above

-Removed ‘9/9’ and replaced with ‘all cases’

Tables 2-4 should be combined
-The tables have been combined into a single Table 2 and references within the manuscript edited to reflect the change

Discussion:

First paragraph: Need to explicitly state that, while persistent lameness was typical, lameness had improved.

-Added sentence to state that lameness had improved in 9/14 cases

Line 333: Do not report % - numbers are too small therefore % are misleading.

-Percentages have been removed

Line 359: Was mechanical in nature

-edited sentence to say ‘was mechanical in nature’

Line 375: A little too speculative; consider deleting this paragraph (manuscript is already very long)

-paragraph was removed

Line 391-393: this needs to be stated in the results

-This sentence was moved to the results and reworded to be included in the Discussion

Line 398-404: Consider deleting from “To increase the accuracy…” to the end of the paragraph.

-This section has been removed along with the references that were stated in the section

Reviewer #2:

I wish to thank the authors for making the previously suggested modifications. I have read the manuscript, and although it reads well, I still have a few minor comments to address prior to acceptance. These are listed below, but I don’t expect to see the manuscript again prior to acceptance so I want to wish you all the best with your future research.
Please note that these are only suggestions and I shall not be disappointed if they are not actioned.

Within the abstract, is it possible to have both the n and a % value? So lines 40. 41 and 42

-The percentages have been removed

Line 110- there is only 21 here, not the 22 which were included, Can you please add in the 22nd?

-This sentence is correct in that there were only 21 dogs- but one case had bilateral elbows operated making it 22 total procedures

Line 116- again there are only 20 here, can you add in the other two please?

-Recounted all patients and appropriately categorized. Number is now 22 including the bilateral case

Line 143- completed, the bone graft (reword)

-Did not add the word ‘the’

Line 144- grafted, an autogenous …(reword)

-Added the word ‘an’

Line 153- screws rather than screw

-Edited to make word plural

Line 165- did not receive a bone graft (reword)

-Added ‘a’

Line 179- non union, and deep SSI (reword)

-Added ‘and’

Line 228- screw, and humeral fissure (reword)

-Added ‘and’
